# Iatrogenic Water Intoxication After Intravenous Infusion of Sterile Water: A Rare and Preventable Case

**DOI:** 10.7759/cureus.88253

**Published:** 2025-07-18

**Authors:** Sudeep R Gorla, Tyler Zander, Melissa A Kendall, Christian Schuetz, Rachel L Wolansky

**Affiliations:** 1 Department of Surgery, University of South Florida Morsani College of Medicine, Tampa, USA; 2 Department of Surgery, Florida State University College of Medicine, Tallahassee, USA

**Keywords:** electrolyte imbalance, iatrogenic complications, intravenous fluid therapy, medical spa regulations, water intoxication

## Abstract

Elective intravenous infusion therapies are increasingly offered in outpatient settings, often without adequate physician oversight or protocols for safety. We describe a case of accidental iatrogenic injury following an intravenous infusion at a medical spa. An elderly woman with a solitary kidney presented to the ED with chest tightness, acute shortness of breath, and headache shortly after receiving an intravenous vitamin infusion in a liter of sterile water, followed by half a liter of normal saline. She rapidly deteriorated, developing flash pulmonary edema and pulseless electrical activity arrest. Following resuscitation, she developed severe metabolic acidosis, hemoconcentration, and progressive anuric renal failure necessitating dialysis. Her hospital course was further complicated by ischemic colitis and perforation, requiring colectomy and ostomy creation. Contributing factors in the mechanism of injury included suspected hemolysis, high-dose vitamin C infusion, and diminished renal reserve. This case underscores the catastrophic impact of inappropriate intravenous fluid administration and highlights the urgent need for increased regulatory oversight in non-hospital settings.

## Introduction

Water intoxication, or dilutional hyponatremia, is a life-threatening condition caused by excessive water intake that overwhelms the kidneys’ ability to excrete free water. This results in hypo-osmolality, cerebral edema, and potentially fatal neurological dysfunction [1]. Although most commonly associated with psychogenic polydipsia in psychiatric patients, it also occurs in endurance athletes, individuals using ecstasy, and hospitalized patients who receive inappropriate fluid therapy [2].

Iatrogenic hyponatremia is another underrecognized cause, frequently seen in hospitalized patients receiving excessive intravenous fluids or postoperative fluid replacement without proper sodium monitoring [3]. Studies suggest that iatrogenic hyponatremia accounts for a significant proportion of hospital-acquired electrolyte disturbances, particularly in cases involving syndrome of inappropriate antidiuretic hormone (SIADH) release, kidney dysfunction, or aggressive hypotonic fluid administration [4]. Despite its well-documented pathophysiology, water intoxication remains underdiagnosed, especially in high-risk populations. Early recognition, routine electrolyte monitoring, and cautious fluid administration are essential to prevent neurological deterioration and sudden death. This case draws attention to preventable iatrogenic complications resulting from unregulated intravenous fluid therapies.

The increasing popularity of elective intravenous hydration and vitamin infusions has led to their widespread availability outside traditional healthcare settings, including concierge medicine practices, medical spas, and intravenous therapy lounges. While many of these services are administered by licensed healthcare providers, protocols for fluid composition, electrolyte monitoring, and patient evaluation can vary significantly [5]. Unlike hospital-based intravenous therapy, where fluid administration is typically guided by laboratory values and clinical status, these outpatient services may lack routine screening and real-time monitoring, increasing the risk of electrolyte imbalances and iatrogenic complications [6]. Additionally, in clinical settings, intravenous medications are compounded or pre-mixed by licensed pharmacists using isotonic solutions such as saline or dextrose, in adherence to United States Pharmacopeia (USP) standards. These practices provide safeguards that may not be present in outpatient settings. In particular, free water infusions without appropriate electrolyte supplementation can lead to acute hyponatremia, a condition that may cause neurological deterioration, pulmonary edema, and cardiac instability.

In this case, we describe a severe episode of iatrogenic water intoxication, highlighting the challenges and dangers of fluid management. It also serves as a cautionary tale about the consequences of inadvertently administering inappropriate intravenous fluids. This case reminds us that even seemingly benign intravenous therapies can lead to devastating outcomes.

## Case presentation

A female patient with a history of hypothyroidism, hyperlipidemia, and a prior left radical nephrectomy presented to the ED with acute dyspnea, chest tightness, and headache after receiving an intravenous vitamin infusion at an alternative medicine clinic. The reported infusion consisted of one liter of sterile water with magnesium and B vitamins, followed by half a liter of normal saline infused with 15 g of vitamin C. Within hours of receiving the infusion, the patient developed dark urine and shortness of breath, prompting her transport to the hospital.

On arrival at the ED, she was hypertensive with a blood pressure of 175/101 mmHg, a heart rate of 85 beats per minute, a respiratory rate of 18 breaths per minute, and an oxygen saturation of 95% on room air. She was initially alert and conversant but expressed an impending sense of doom to the medical staff. She then rapidly deteriorated, developing worsening hypoxia and hemoptysis with frothy, blood-tinged sputum. Soon after, she suffered pulseless electrical activity (PEA) cardiac arrest.

Three rounds of cardiopulmonary resuscitation were performed, and she received two one-milligram doses of epinephrine, five grams of calcium gluconate, and two 50-mEq ampules of sodium bicarbonate. Return of spontaneous circulation was achieved, and she was intubated for acute respiratory failure. A central line, an arterial line, and a dialysis catheter were placed for ongoing resuscitation and hemodynamic monitoring. 

Initial laboratory analysis showed a pH of 6.94, a pCO₂ of 59 mmHg, and a lactate level of 12.2 mmol/L. The electrolyte profile included a sodium level of 154 mmol/L, a potassium level of 4.4 mmol/L, and a calcium level of 13.7 mg/dL. The patient’s troponin level was initially 84 ng/L but increased to 2,000 ng/L over two to three hours following the initial measurement. Her white blood cell count was 18,500, and hemoglobin was 17.9 g/dL. She was anuric, with an initial creatinine of 1.17 mg/dL and a blood urea nitrogen level of 18 mg/dL (Table 1). Chest radiography revealed diffuse pulmonary opacities indicative of multifocal pneumonia or acute respiratory distress syndrome (ARDS). She was admitted to the ICU from the ED for further management.

**Table 1:** Key laboratory findings upon initial admission to ED. Reference value range as given by treating facility BUN: blood urea nitrogen; WBC: white blood cell count; pCO₂: partial pressure of carbon dioxide; Na⁺: sodium ion concentration; K⁺: potassium ion concentration; HCO_3_⁻: serum bicarbonate concentration; Ca^2^⁺: calcium ion concentration; mmHg: millimeters of mercury

Test	Patient’s Value	Reference Range
Arterial blood pH	6.94	7.35–7.45
pCO₂ (mmHg)	59	35–45
Lactate (mmol/L)	12.2	0.9–1.8
Sodium (Na⁺) (mmol/L)	154	136–145
Potassium (K+) (mmol/L)	4.4	3.6–5.0
Serum CO_2_ (HCO_3_-) (mEq/L)	12.7	22–26
BUN (mg/dL)	18	8–23
Creatinine (mg/dL)	1.17	0.55–1.02
Calcium (Ca^2^⁺) (mg/dL)	13.7	8.3–10.2
Troponin (ng/L)	84	<60
WBC (×10^3^/L)	18.5	4.5–11
Hemoglobin (g/dL)	17.9	11.6–16.1

Upon ICU admission, the patient remained hemodynamically unstable with episodes of paroxysmal atrial fibrillation and required vasopressor support. She also required mechanical ventilation using ARDS-protective settings, including a fraction of inspired oxygen (FiO₂) of 100%, a positive end-expiratory pressure of 18 cm H₂O, and a tidal volume of 400 mL. Ceftriaxone was administered for suspected aspiration pneumonia. The patient remained anuric, with worsening metabolic acidosis and electrolyte abnormalities, necessitating the initiation of continuous renal replacement therapy. The patient exhibited signs of metabolic encephalopathy; however, a CT of the head was unremarkable.

**Figure 1:**
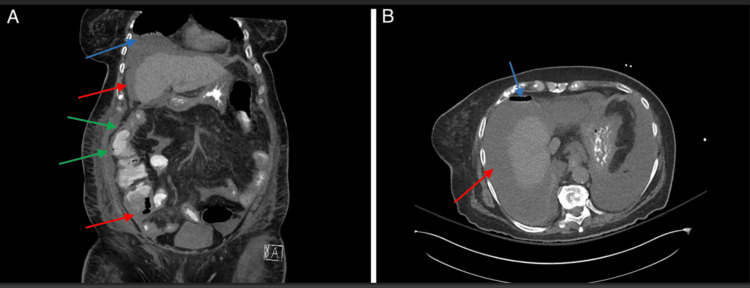
Representative images from CT abdomen/pelvis Pertinent findings include: (A) moderate volume of free fluid (red arrows), pneumoperitoneum (blue arrow), and pneumatosis (green arrows) of the ascending colon; and (B) free fluid (red arrow) and pneumoperitoneum (blue arrow). The presence of pneumatosis intestinalis and free air indicated colonic ischemia with perforation, corresponding clinically to the patient’s acute abdominal pain, hemodynamic instability, and need for emergent surgical intervention. These findings reflect late-stage complications of hypoperfusion and vasopressor-associated ischemia in the setting of multiorgan system failure.

On hospital day eight, the patient was extubated but had a persistent altered mental status, which gradually resolved with frequent reorientation and correction of underlying metabolic disturbances. She later developed acute abdominal pain and worsening hemodynamic instability. On hospital day 16, a CT scan of the abdomen was obtained, which revealed suspected pneumatosis intestinalis and pneumoperitoneum (Figure 1). She was taken to the operating room, where she was found to have ischemic colitis with perforation, and she underwent an extended right hemicolectomy with ileostomy creation.

Postoperatively, she had persistent renal failure requiring ongoing hemodialysis. Her hemodynamic status gradually improved, and she was weaned off vasopressors. By hospital day 23, she was deemed medically stable for transfer to a long-term acute care facility for continued dialysis and rehabilitation.

## Discussion

This case represents a rare and extreme instance of iatrogenic water intoxication leading to rapid multiorgan dysfunction. Typically, the primary pathology following free water intoxication is acute hyponatremia, which often develops within hours of excessive oral hypotonic fluid intake. Acute hyponatremia normally presents with predominantly neurologic symptoms, including nausea, headache, confusion, seizures, and, in severe cases, coma and brain herniation due to cerebral edema [1,7]. Pulmonary findings may occur but are usually secondary to neurologic dysfunction or SIADH [8].

Our case diverges from this classical pattern of symptoms. The intravascular infusion of sterile water resulted in an immediate and dangerous osmotic imbalance. As a profoundly hypotonic solution, sterile water rapidly shifted into the intracellular compartment, leading to cellular swelling, hemolysis, and endothelial injury [1]. The red cell lysis and capillary leak that followed likely triggered systemic inflammation, vascular collapse, and metabolic derangement. This mechanism contrasts with the more gradual intoxication seen in psychiatric polydipsia or 3,4-methylenedioxymethamphetamine (MDMA) use, where the sodium decline develops over hours to days and primarily causes neurologic symptoms [1,7].

The patient’s laboratory findings reflect a rapidly evolving state of systemic collapse initiated by osmotic injury. The severe metabolic acidosis from hemolysis-induced endothelial dysfunction and circulatory failure led to widespread tissue hypoxia and impaired perfusion. Respiratory compensation was inadequate, suggesting evolving pulmonary compromise and early ventilatory insufficiency. The elevated sodium level, though unexpected after hypotonic fluid exposure, likely reflects an intracellular fluid shift compounded by impaired renal clearance from her solitary kidney. This was further exacerbated by the administration of a corrective isotonic saline bolus at the medspa and resuscitative medications shortly after arrival to the ED. This pattern has been described in acute hypotonic injuries followed by sodium-containing infusion, where initial hyponatremia may be obscured by the time of testing [7].

The concentration of hemoglobin and leukocytosis further supports hemoconcentration and an acute systemic inflammatory response. Her potassium levels remained within normal limits, which is contradictory to what is expected in the setting of hemolysis. However, these values must be interpreted cautiously, considering the presence of severe acidosis and evolving renal dysfunction. Possible upregulation of the renin-angiotensin-aldosterone system due to physiologic stress and cardiac arrest leads to greater sodium reabsorption and potassium excretion, which may provide a partial explanation for the electrolyte values [9]. Troponin elevation suggests cardiac strain or demand ischemia in the context of global hypoperfusion. Hypercalcemia, although multifactorial, may be partly attributed to cellular injury, calcium-containing resuscitation, or impaired renal clearance.

Within the cascade of worsening organ dysfunction, the development of acute kidney injury in this patient was multifactorial in origin. The cardiogenic shock following pulseless electrical activity (PEA) arrest, hemolysis-induced renal injury due to hemoglobinuria, and reduced renal reserve due to a solitary kidney were likely contributors (Table 2). Moreover, the high dose (15 g) of intravenous vitamin C likely induced hyperoxaluria [10]. Ascorbic acid is not inherently nephrotoxic. However, when metabolized to oxalate, it may worsen preexisting tubular injury, particularly in the setting of hemolytic or ischemic stress [10].

**Table 2:** Summary of key pathophysiological mechanisms and resulting clinical outcomes IV: intravenous; ARDS: acute respiratory distress syndrome; AKI, acute kidney injury; PEA: pulseless electrical activity

Event	Pathophysiological Mechanism	Clinical Consequence(s)
IV infusion of sterile water	Extreme hypotonicity → osmotic shift into cells	Hemolysis, endothelial injury
Hemolysis	Free hemoglobin → tubular injury	Acute kidney injury
Capillary leak + inflammation	Endothelial dysfunction	Pulmonary edema, ARDS
High-dose vitamin C (15g)	Oxalate accumulation in tubules	Hyperoxaluria, worsening AKI
PEA arrest + vasopressors	Global ischemia + splanchnic hypoperfusion	Ischemic colitis, perforation
Preexisting solitary kidney	Diminished renal reserve	Rapid anuria, dialysis dependency

Notably, the patient developed flash pulmonary edema before her PEA arrest. Although she did not receive a blood transfusion, the pathophysiology shares important parallels with the progression seen in transfusion-associated circulatory overload (TACO) and transfusion-related acute lung injury (TRALI). In TACO, excessive fluid administration in patients with existing renal impairments can exceed pulmonary capillary pressure, leading to hydrostatic edema, similar to this case [11]. TRALI, by contrast, results in noncardiogenic edema driven by increased capillary permeability due to inflammation-mediated endothelial cell damage [11]. In this case, the infusion of hypo-osmotic fluid likely triggered elements of both hydrostatic and permeability-mediated pulmonary injury. This dual mechanism of injury overwhelmed pulmonary barrier integrity and gas exchange capabilities, resulting in pathophysiology consistent with hypoxic arrest and progression to ARDS [12].

Complications such as ARDS, ischemic colitis, and cardiac arrest, though devastating, were downstream effects of the initial fluid imbalance (Table 2). The use of vasopressors to manage shock after cardiac arrest likely contributed to bowel ischemia and subsequent perforation due to splanchnic hypoperfusion in the watershed areas of the colon. Additionally, the patient’s status as an elderly female with multiple comorbidities, including a solitary kidney, hypertension, and hyperlipidemia, likely limited her ability to mount an effective physiological response. These factors suggest underlying vascular disease and a reduced compensatory reserve, which may have exacerbated the severity of her decline. The vascular-centric nature of her deterioration, compared to the classic neurologic sequelae of water intoxication, supports the conclusion that sterile water, when administered intravenously inappropriately, acts not merely as a hypotonic agent but as an intravascular toxin. This has been documented in prior cases of accidental hospital infusions, where even small volumes led to hemolysis and death [13].

Despite the growing popularity of medical spas, this case underscores the serious risks associated with performing medical infusions outside regulated healthcare environments. Although licensed providers are technically required to supervise treatments in many states, enforcement is often inconsistent. Intravenous therapies are frequently administered by nonphysician staff with limited formal training in fluid management [4]. In this case, the use of sterile water reflects a lapse in both clinical judgment and oversight. Furthermore, patients receiving infusions in these settings are rarely monitored with the same rigor as in hospitals, where continuous vital sign monitoring, electrolyte screening, and rapid emergency intervention are standard practice [14]. As this patient’s experience demonstrates, the absence of established protocols and accountability structures can result in life-threatening consequences. There is a clear need for stronger regulatory frameworks and standardized guidelines to ensure safety in elective intravenous therapy services.

To improve medspa safety, outpatient infusion practices should adopt protocols commonly used in hospitals. In clinical settings, intravenous therapies are compounded by licensed pharmacists in sterile environments according to standards set by the United States Pharmacopeia (USP). These treatments are administered under physician supervision with close patient monitoring. In contrast, the FDA has reported serious complications resulting from unsanitary compounding and unregulated administration of intravenous therapies in hydration clinics and medspas [15]. Medspas should adopt USP standards that mandate aseptic technique, ISO-classified cleanrooms, and proper labeling of fluids. While not all outpatient facilities can replicate hospital infrastructure, applying core principles such as pharmacist oversight and individualized patient assessment can reduce iatrogenic risks associated with intravenous therapy.

## Conclusions

This case highlights the life-threatening consequences of routine intravenous free water administration, serving as a powerful reminder that even commonly used intravenous therapies can lead to catastrophic outcomes when improperly administered, particularly outside traditional hospital settings. An extremely hypotonic solution can cause immediate osmotic disturbances, including hemolysis, flash pulmonary edema, and cardiac arrest, as observed in this patient. Unlike classic water intoxication, which typically presents with neurologic symptoms due to hyponatremia, this patient’s course was dominated by vascular collapse, multiorgan dysfunction, and irreversible renal failure. Her outcome underscores the critical importance of understanding fluid tonicity. It also emphasizes the need for standardized protocols, clinical education, and regulatory enforcement in non-hospital intravenous infusion settings to prevent similar occurrences in the future.
